# Combined effects of ivabradine with dobutamine or levosimendan in isolated perfused hearts

**DOI:** 10.1186/cc12166

**Published:** 2013-03-19

**Authors:** D Konrad, R Vicenzi-Moser, M Vicenzi, U Aldenhoff, E Schwarzl, W Toller

**Affiliations:** 1 Medical University Graz, Austria

## Introduction

Dobutamine and levosimendan improve cardiac contractility in patients with heart failure, septic cardiomyopathy or cardiac surgery. Tachycardia in these cases is undesired because of aggravating ischemia. Ivabradine, a selective sinus node inhibitor, does not affect contractility. A combination of ivabradine with positive inotropic drugs might be favorable. Thus, we compared the cardiac effects of dobutamine or levosimendan alone, and combined with ivabradine using the Langendorff method of isolated perfused hearts.

## Methods

Isolated guinea pig hearts (*n = *37) were perfused with incremental doses of dobutamine (10 nM to 10 μM) or levosimendan (30 nM to 10 μM) either alone or combined with 3 μM ivabradine. Heart rate (HR), left ventricular pressures, contractility (+dLVP/dt) and relaxation (-dLVP/dt) were recorded. Data for each drug (dobutamine or levosimendan) were analyzed by two-way ANOVA for repeated measures including the two main effects of ivabradine and drug dose and their interaction. Data are reported as mean ± standard deviation.

## Results

Ivabradine decreased the HR from 223 ± 18 to 196 ± 15 bpm (*P <*0.05). Contractility and relaxation remained unchanged. Ivabradine reduced the positive chronotropic effect at all doses of dobutamine (10 nM: 232 ± 37 vs. 187 ± 19, 100 nM: 265 ± 37 vs. 211 ± 40, 1 μM: 316 ± 35 vs. 250 ± 39, 10 μM: 320 ± 33 vs. 235 ± 40 bpm; *P <*0.05). It shifted the maximum positive inotropic action of dobutamine to lower dose ranges (100 nM: 2,924 ± 841 vs. 2,978 ± 955, 300 nM: 3,743 ± 925 vs. 4,795 ± 1,298, 1 μM: 4,138 ± 935 vs. 4,896 ± 1,861 mmHg/second; *P <*0.05). A comparable shift was seen for relaxation (100 nM: -2,178 ± 686 vs. -2,520 ± 742, 300 nM: -2,615 ± 726 vs. -3,150 ± 888, 1 μM: -2,903 ± 752 vs. -2,972 ± 967 mmHg/second; *P <*0.05). Levosimendan increased the HR only at high doses. With ivabradine, no positive chronotropic effect of levosimendan was observed (100 nM: 185 ± 30 vs. 162 ± 22, 1 μM: 208 ± 28 vs. 166 ± 29, 10 μM: 242 ± 27 vs. 168 ± 36 bpm; *P <*0.05). Ivabradine attenuated the positive inotropic effect of levosimendan (100 nM: 2,303 ± 303 vs. 1,737 ± 262, 1 μM: 2,977 ± 481 vs. 1,940 ± 449, 10 μM: 3,480 ± 941 vs. 2,189 ± 542 mmHg/ second; *P <*0.05) but did not significantly alter its lusitropic effect.

## Conclusion

Addition of ivabradine to dobutamine attenuates its chronotropic actions without diminishing its inotropic effects. A combination of levosimendan with ivabradine does not seem to provide benefit. Clinical studies are necessary to confirm these experimental results.

